# BOT-4-one attenuates NLRP3 inflammasome activation: NLRP3 alkylation leading to the regulation of its ATPase activity and ubiquitination

**DOI:** 10.1038/s41598-017-15314-8

**Published:** 2017-11-08

**Authors:** Do-Wan Shim, Woo-Young Shin, Sang-Hyeun Yu, Byung-Hak Kim, Sang-Kyu Ye, Sushruta Koppula, Hyung-Sik Won, Tae-Bong Kang, Kwang-Ho Lee

**Affiliations:** 1 Department of Applied Life Science, Graduate School, Konkuk University, Chungju, Korea; 2 Department of Pharmacology, Seoul National University College of Medicine, Seoul, Republic of Korea; 3 CYTUS H&B Co., Ltd, Cheongju, Republic of Korea; 4 Department of Biotechnology, Research Institute of Inflammatory Disease, Konkuk University, Chungju, Korea

## Abstract

The ATPase activity of NLRP3 has pivotal role in inflammasome activation and is recognized as a good target for the development of the NLRP3 inflammasome-specific inhibitor. However, signals in the vicinity of the ATPase activity of NLRP3 have not been fully elucidated. Here, we demonstrate NLRP3 inflammasome-specific action of a benzoxathiole derivative, BOT-4-one. BOT-4-one exhibited an inhibition of NLRP3 inflammasome activation, which was attributable to its alkylating capability to NLRP3. In particular, the NLRP3 alkylation by BOT-4-one led to an impaired ATPase activity of NLRP3, thereby obstructing the assembly of the NLRP3 inflammasome. Additionally, we found that NLRP3 alkylators, including BOT-4-one, enhance the ubiquitination level of NLRP3, which might also contribute to the inhibition of NLRP3 inflammasome activation. Finally, BOT-4-one appeared to be superior to other known NLRP3 alkylators in inhibiting the functionality of the NLRP3 inflammasome and its resulting anti-inflammatory activity was confirmed *in vivo* using a monosodium urate-induced peritonitis mouse model. Collectively, the results suggest that NLRP3 alkylators function by inhibiting ATPase activity and increasing the ubiquitination level of NLRP3, and BOT-4-one could be the type of NLRP3 inhibitor that may be potentially useful for the novel development of a therapeutic agent in controlling NLRP3 inflammasome-related diseases.

## Introduction

Inflammasomes are intracellular multiprotein complexes that activate inflammatory cascades in response to infectious pathogens and damage-associated molecular patterns (DAMPs). The activation of inflammasomes triggers caspase-1 activation, which subsequently cleaves pro-IL-1β and pro-IL-18 for their maturation into active forms^1^. Additionally, canonical inflammasome activation by caspase-1 as well as noncanonical inflammasome activation through caspase-4, 5, and 11 can lead to cell death called pyroptosis. As such an important component of innate immunity, inflammasome functionalities are also associated with human health and the development of various inflammation-related disorders, including autoinflammatory and autoimmune diseases^2^. Component molecules that organize the inflammasomes are therefore considered to be promising targets for anti-inflammatory therapies.

An inflammasome is assembled with central sensor molecules connecting to caspase-1 via the adaptor protein ASC^3^. In particular, the sensor molecules determine the type of inflammasome, whereas the other components are common to all inflammasomes. Several sensor molecules have been identified, including absent in melanoma 2 (AIM2), IFNγ-inducible protein 16 (IFI16) and various NOD-like receptor (NLR) subsets^4^. Among them, the NLRP3 inflammasome in the NLR family represents one of the most well-characterized inflammasomes, of which excessive and persistent activation can induce metabolic disorders, such as type 2 diabetes, gout and atherosclerosis^5^. In particular, like other NLR subsets, NLRP3 has a central nucleotide-binding domain called NACHT, which is responsible for the ATP-dependent oligomerization processes^6,7^. Consequently, it has been found that diminishing ATPase activity decreases NLRP3 self-oligomerization and its intermolecular association with ASC, which constitutes one of the critical steps for inflammasome activation^7,8^. However, details of ATPase activity still remain elusive, as its structural information is not available yet and other signals related to the ATPase and/or oligomerization processes have not been fully elucidated.

Recently, several chemical compounds have been searched for as promising anti-inflammatory agents that directly inhibit the activation of the NLRP3 inflammasome. Among these, the synthetic IκB kinase-β inhibitor Bay11-7082, which was originally developed as an NF-κB pathway inhibitor, was revealed to also selectively inhibit NLRP3 inflammasome activity in an NF-κB-independent manner^9^. Other chemicals, including the Syk kinase inhibitor 3,4-methylenedioxy-β-nitrostyrene (MNS) and some acrylamide derivatives, have also been identified as such NLRP3 inflammasome inhibitors^10,11^. The functional activity of those compounds appear to be critically mediated through the inhibition of the ATPase activity of NLRP3. In addition, all of those chemicals were suggested as most likely acting via covalent linkage to NLRP3. Such an alkylation reaction could probably be achieved by nucleophilic attacks with the reactive residues (such as cysteines) in the target protein^9–11^. Computational analysis predicted the Cys419 residue in the ATPase catalytic pocket of NLRP3 as the specific target site for the acrylamide derivatives^11^. In this context, the inhibition of the NLRP3 ATPase activity through NLRP3 alkylation can be regarded as an ideal target for developing specific inhibitors of the NLRP3 inflammasome^12^.

**Figure 1:**
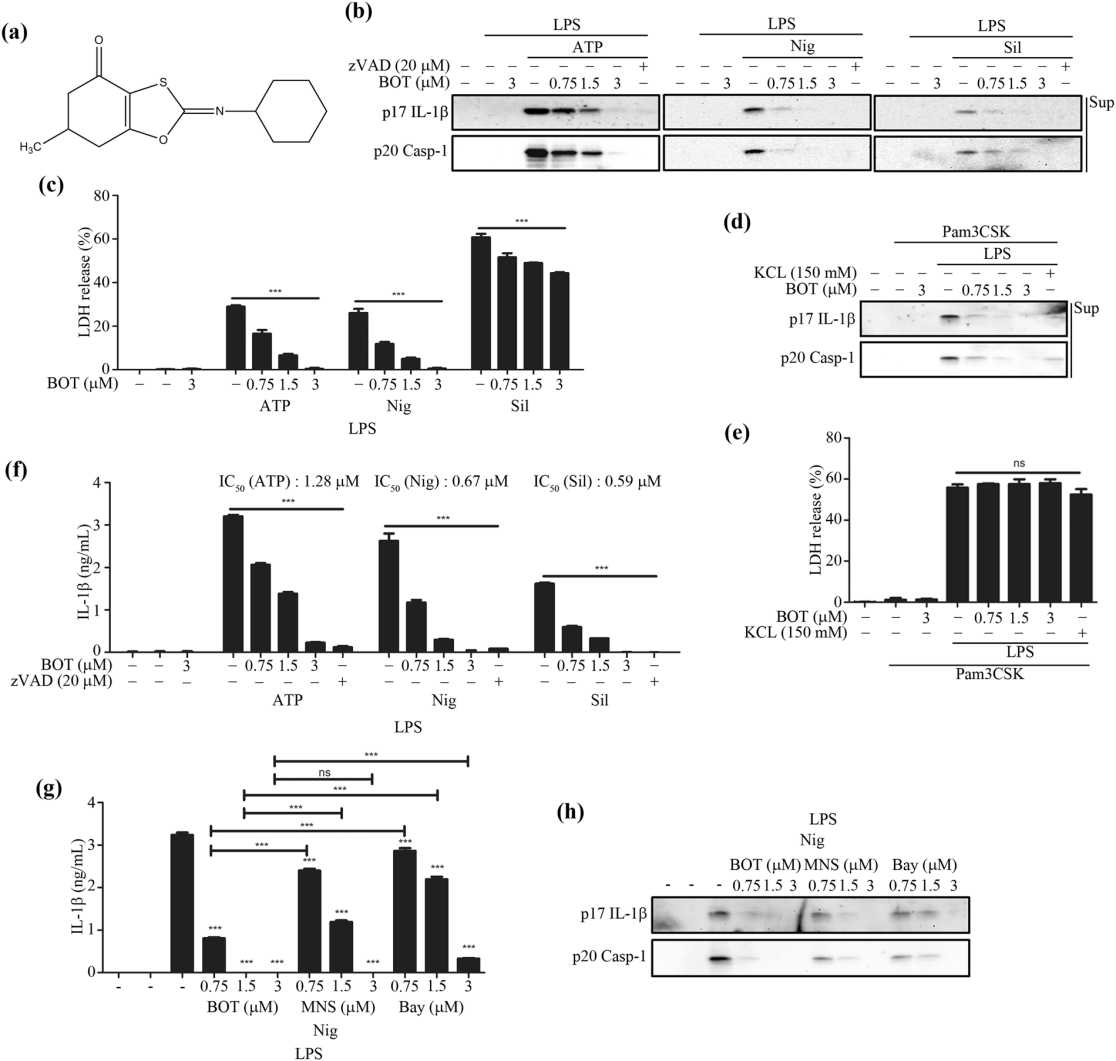
BOT-4-one inhibits NLRP3 inflammasome activation. (**a**) The molecular structure of BOT-4-one. LPS-primed BMDMs were treated with BOT-4-one or zVAD for 1 h and then stimulated with ATP, nigericin (Nig), or silica crystals (Sil). The caspase inhibitor zVAD was employed as a positive control for the general inhibition of inflammasome activations. (**b**) IL-1β (p17) and cleaved caspase-1 (Casp-1) in the supernatants (Sup) were analyzed by immunoblot. Full-length blots/gels are presented in Supplementary Fig. S9a. (**c**) LDH release in cell supernatants was measured by LDH assay. Pam3CSK4-primed BMDMs were treated with BOT-4-one or KCl for 1 h and then transfected with LPS. KCl was used as a positive control for the NLRP3 inflammasome-specific inhibition. The supernatants were analyzed by immunoblot (**d**) and LDH assay (**e**). Full-length blots/gels are presented in Supplementary Fig. S9b. (**f**) LPS-primed BMDMs were treated with BOT-4-one or zVAD for 1 h and then stimulated with ATP, nigericin (Nig), or silica crystals (Sil). IL-1β secretion in the cell supernatants was measured by ELISA. LPS-primed BMDMs were treated with BOT-4-one, MNS, or Bay11-7082 (Bay) for 1 h, and then stimulated with nigericin (Nig). The supernatants were analyzed by ELISA (**g**) and immunoblot (**h**). Full-length blots/gels are presented in Supplementary Fig. S9c. The data represent the mean ± SEM of three independent experiments; ***p < 0.001; ns: non-significant.

Many benzoxathiole derivatives are known to possess various pharmacological properties, including anti-microbial, cytostatic, anti-psoriatic, and anti-mycotic activities^13–15^. A novel benzoxathiole derivative, BOT-4-one (2-cyclohexylimino-6-methyl-6,7-dihydro-5*H*-benzo[1,3]oxathiol-4-one; Fig. 1a), which can exhibit anti-cancer effects^16^, has been recently documented as also exhibiting an immunomodulatory activity that alleviates 2,4,6-trinitrochlorobenzene (TNCB)-induced dermatitis and collagen-induced arthritis^17,18^. For these anti-inflammatory activities, BOT-4-one has been validated as an NF-κB signaling inhibitor that functions through alkylating target proteins. The kinase domain of IKKβ was identified as the most likely target of alkylation by BOT-4-one in the NF-κB pathway and a plausible nucleophilic addition with Cys179 residue could be modeled as the corresponding alkylation reaction^17^. Therefore, in our study, we aimed to verify the NF-κB pathway-independent potential of BOT-4-one for selective alkylation targeting the ATPase activity of NLRP3 to inhibit the activation of the NLRP3 inflammasome, as in the case of Bay11-7082. This work also investigated whether the regulation of NLRP3 ubiquitination, as another signal in the vicinity of the ATPase and/or oligomerization processes, could be involved in the functionalities of NLRP3 alkylators.

## Results

### BOT-4-one potently inhibits the activation of the NLRP3 inflammasome

The non-toxic and effective concentration of BOT-4-one was determined by cell viability assays using the murine bone marrow-derived macrophages (BMDMs) and PMA-differentiated THP-1 human leukemic monocytes. As no significant cytotoxicity was observed with BOT-4-one up to 3 μM in both BMDMs and THP-1 cells (Supplementary Fig. S1), 3 μM of the BOT-4-one treatment was employed in subsequent experiments. To assess the effects of BOT-4-one on the activation of the canonical NLRP3 inflammasome, LPS-primed BMDMs were treated with NLRP3 inflammasome activators in the presence or absence of BOT-4-one. The results showed that BOT-4-one significantly inhibited in a dose-dependent manner the NLRP3 inflammasome-mediated outcomes, including IL-1β secretion, caspase-1 cleavage, and lactate dehydrogenase (LDH) release (Fig. 1b and c). Those inhibitory effects appeared to be evident in PMA-differentiated THP-1 cells as well (Supplementary Fig. S2a–c).

The effects of BOT-4-one were then examined on noncanonical inflammasomes, which were activated by LPS transfection into the Pam3CSK4-primed BMDMs. It is known that the activation of noncanonical inflammasomes by Gram-negative bacteria and intracellular LPS results in caspase-11-dependent pyroptosis, for which NLRP3 is dispensable, and in IL-1β secretion, which essentially requires both NLRP3 and caspase-11^19,20^. As expected, a dose-dependent inhibition of the NLRP3-mediated IL-1β secretion by BOT-4-one was observed (Fig. 1d), whereas the caspase-11-dependent LDH release was not affected (Fig. 1e). Collectively, the results indicated that BOT-4-one inhibits the activation of both canonical and noncanonical NLRP3-inflammasomes.

Judging from the inhibitory effect on IL-1β secretion, the estimated IC_50_ values of BOT-4-one were approximately 1.28, 0.67, and 0.59 μM against ATP-, nigericin-, and silica crystal-induced activation of the NLRP3 inflammasome, respectively (Fig. 1f), indicating a highly potent activity of BOT-4-one. In addition, BOT-4-one exhibited more potent inhibitions of the NLRP3 inflammasome-mediated IL-1β secretion and caspase-1 activation than MNS and Bay11-7082 (Fig. 1g and h), which are known as NLRP3-alkylating agents and widely used as NLRP3 inflammasome inhibitors^9,10^. Hence, BOT-4-one could be evaluated as a highly potent inhibitor of NLRP3 inflammasome, superior to the other known compounds MNS and Bay11-7082.

### The inhibitory action of BOT-4-one is specific to the NLRP3 inflammasome

**Figure 2:**
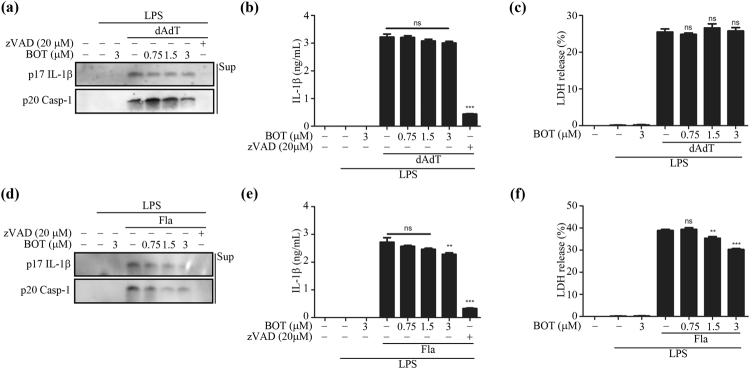
Effect of BOT-4-one on AIM2 and NLRC4 inflammasome activation. LPS-primed BMDMs were treated with BOT-4-one or zVAD for 1 h and then transfected with poly(dA:dT) (dAdT) or flagellin (Fla). (**a** and **d**) Supernatants were analyzed by immunoblot. IL-1β secretion (**b** and **e**) and LDH release (**c** and **f**) in the cell supernatants were measured by ELISA and LDH assay, respectively. Full-length blots/gels are presented in S Fig. 9d and e. The data represent the mean ± SEM of three independent experiments; **p < 0.01; ***p < 0.001; ns: non-significant.

To examine whether BOT-4-one activity is specific to NLRP3 inflammasome, its effects were monitored on other types of inflammasomes including AIM2 and NLRC4. The LPS-primed BMDMs were transfected with the AIM2 inflammasome activator poly(dA:dT)^21^ and the NLRC4 inflammasome activator flagellin^22^, respectively, in the presence or absence of BOT-4-one. The results showed that BOT-4-one did not inhibit the AIM2 inflammasome-mediated IL-1β secretion, caspase-1 cleavage, and LDH release (Fig. 2a–c). No significant effect on the IL-1β secretion was also validated in the PMA-differentiated THP-1 cells (Supplementary Fig. S2d).

In the case of NLRC4 inflammasome activation, a modest suppression of IL-1β and caspase-1 maturation by BOT-4-one was observed at the highest concentration (3 µM) tested (Fig. 2d–f), although a clear dose-dependency could not be supported. However, this marginal inhibition could not be verified as a direct action of BOT-4-one on the NLRC4 inflammasome, because it has recently been suggested that the activation of the NLRC4 inflammasome is only marginally associated with NLRP3 (refer to Discussion)^23^. Even though BOT-4-one has a direct inhibition of the NLRC4 inflammasome, the observed effects were slight compared with NLRP3 inflammasomes to be considered as specific ones, and no other cognate processes such as ASC speck formation were significantly inhibited (refer to the following sections). Therefore, taken together, BOT-4-one could be regarded as a specifically potent inhibitor of the NLRP3 inflammasome with no or little influence on the AIM2 and NLRC4 inflammasomes.

### BOT-4-one hinders NLRP3-mediated ASC speck formation

**Figure 3:**
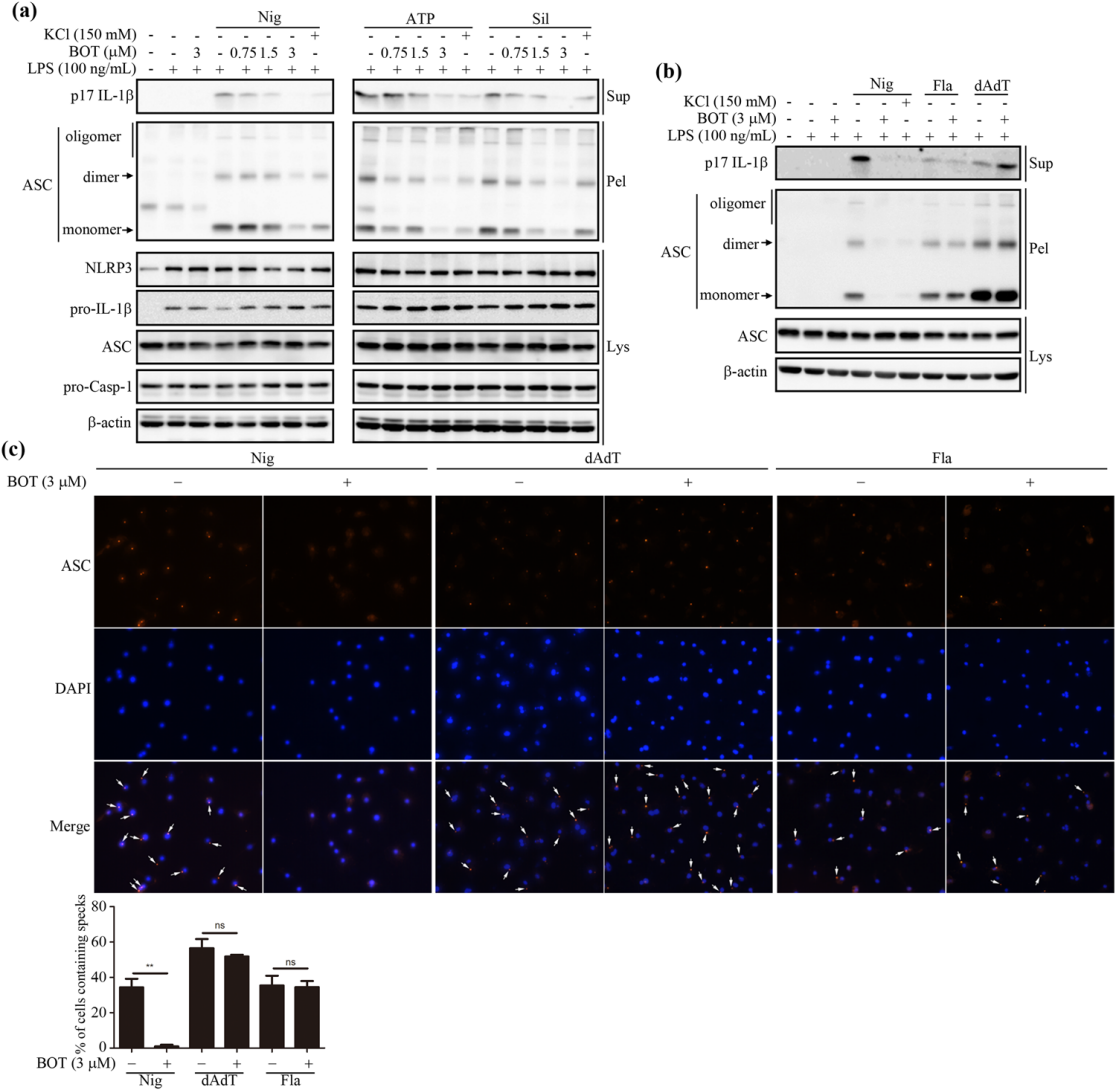
BOT-4-one blocks NLRP3-dependent ASC oligomerization. (**a**) LPS-primed BMDMs were treated with BOT-4-one at different concentrations or KCl for 1 h and then stimulated with nigericin (Nig), ATP, or silica crystals (Sil). (**b**) LPS-primed BMDMs were treated with BOT-4-one or KCl for 1 h and then stimulated with nigericin (Nig), poly(dA:dT) (dAdT), or flagellin (Fla). (**a** and **b**) ASC oligomerization in cross-linked cytosolic pellets was analyzed by immunoblot. Full-length blots/gels are presented in Supplementary Fig. S10a and b. (**c**) Representative immunofluorescence images of ASC speck formation are shown in LPS-primed BMDMs stimulated with nigericin (Nig), poly(dA:dT) (dAdT), or flagellin (Fla) in the presence or absence of BOT-4-one. The data represent the mean ± SEM of three independent experiments; **p < 0.01; ns: non-significant.

ASC is one of the major components of inflammasomes. As the oligomerization of ASC that leads to the formation of insoluble specks called ‘pyroptosomes’ is critically involved in the activation of inflammasomes, its inhibition is known to prevent inflammasome activation^24,25^. To examine whether BOT-4-one prevented ASC speck formation, the cells stimulated by NLRP3 inflammasome activators (nigericin, ATP, and silica crystals,) were lysed with Triton X-100 detergents and the resulting pellets were subjected to chemical cross-linking, followed by immunoblots detecting ASC. The detection of cross-linked ASC indicated that the insoluble specks of ASC oligomers were contained in the Triton X-100-insoluble fraction as expected^7^ and the results also showed that the cytosolic pellets of BOT-4-one-treated cells contained quite lower amounts of ASC specks compared with the LPS-inflammasome activators treated control groups (Fig. 3a and b). In contrast, no significant effects of BOT-4-one were observed for the flagellin- and poly(dA:dT)-stimulated cells, confirming the NLRP3 inflammasome-specific action of BOT-4-one (Fig. 3b). More definitely, the immunofluorescence image that detected ASC specks showed that BOT-4-one specifically suppressed NLRP3-mediated ASC speck formation (Fig. 3c).

### BOT-4-one reduces the insolublization of both NLRP3 and ASC

**Figure 4:**
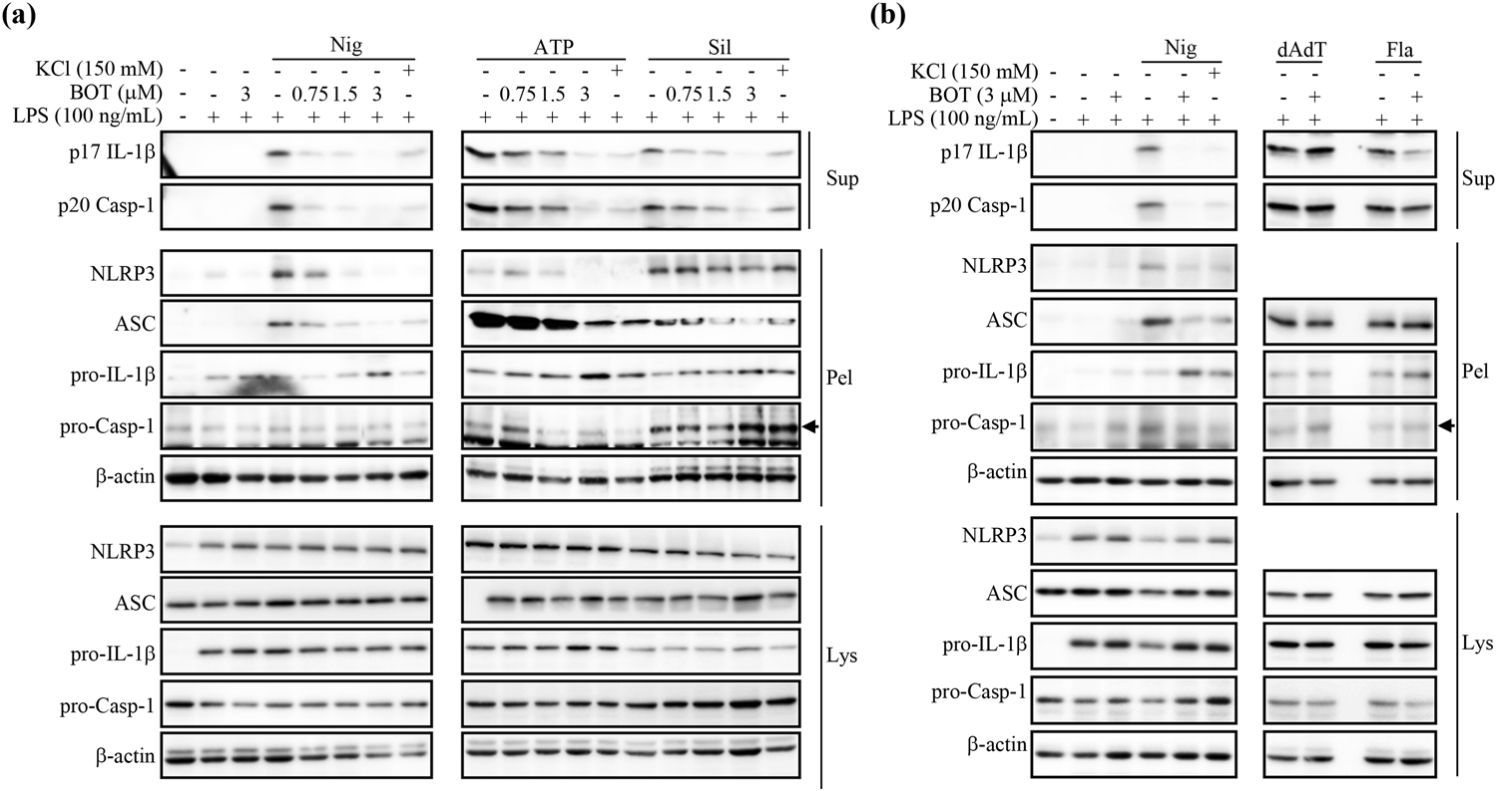
BOT-4-one decreases NLRP3-dependent insolubilization of both NLRP3 and ASC. (**a**) LPS-primed BMDMs were treated with BOT-4-one at different concentrations or KCl for 1 h and then stimulated with nigericin (Nig), ATP, or silica crystals (Sil). (**b**) LPS-primed BMDMs were treated with BOT-4-one or KCl for 1 h and then stimulated with nigericin (Nig), poly(dA:dT) (dAdT), or flagellin (Fla). (**a** and **b**) The cultured supernatants, soluble lysates (Lys), and insoluble pellets (Pel) of Triton × 100 were analyzed by immunoblot. Full-length blots/gels are presented in Supplementary Fig. S11a and b.

As BOT-4-one did not suppress the expression of individual NLRP3 inflammasome components including pro-IL-1β, NLRP3, and ASC in our experimental system (Fig. 3a and b), the observed suppression of ASC speck formation was probably attributable to the prevention of the insolublization of ASC to form the specks. It is known that inflammasome activation is triggered by the self-oligomerization of NLRP3^26,27^, which in turn promotes ASC oligomerization to form the insoluble specks. In addition, NLRP3 as well as ASC translocate into insoluble fractions to form oligomers^7,28–30^, which can also be detected in the insoluble pellets of the Triton X-100-treated cell lysates^24,28,31^. Therefore, we examined the effects of BOT-4-one on the distribution of NLRP3 and ASC into the soluble and insoluble fractions. Upon the stimulation of NLRP3 inflammasome by nigericin, ATP, and silica crystals, suppression of the insoluble ASC by BOT-4-one was detected again in the Triton X-100-insoluble fraction of cell lysates, while the inhibition of IL-1β and caspase-1 secretions was confirmed in the supernatants of the cell culture (Fig. 4). Additionally, the results clearly showed that the NLRP3 in the insoluble fraction was reduced in the presence of BOT-4-one, indicating that BOT-4-one efficiently suppressed the translocation of the NLRP3 into the insoluble fraction. The reduced insolubilization of both NLRP3 and ASC was also validated in the PMA-differentiated THP-1 cells stimulated by nigericin (Supplementary Fig. S3). In contrast, the retained level of the insoluble-fraction of ASC was confirmed again upon the stimulation of AIM2 and NLRC4 inflammasomes by poly(dA:dT) and flagellin in the presence of BOT-4-one (Fig. 4b). The pro-IL-1β levels in the pellet were increased upon nigericin (Nig) treatment in the presence of 3 μM BOT-4-one (Fig. 4a and b). The increased pro-IL-1β in the pellets are possibly due to the accumulation of uncleaved form of pro-IL-1β tranlocated into insoluble fractions in the cells. In summary, the results indicated that the specific action of BOT-4-one in activating the NLRP3 inflammasome was attributable to the suppression of the NLRP3 translocation into an insoluble fraction, which is in turn responsible for the inhibition of ASC oligomerization forming insoluble specks.

For the NLRP3 inflammasome activation, its inducers such as ATP, nigericin, and silica crystals are known to cause the acetylation of α-tublin and the acetylated α-tublin in turn plays an important role in the redistribution of both NLRP3 and ASC^32^. In addition, the loss of mitochondrial membrane potential is also closely related to the NLRP3 inflammasome activation^33^. In the present study, BOT-4-one affected neither the nigericin-induced α-tubulin acetylation (Supplementary Fig. S4a) nor the ATP- and nigericin-induced losses of mitochondrial membrane potential (Supplementary Fig. S4b). Therefore, the direct regulation of NLRP3 was then considered as the most plausible site of the molecular action of BOT-4-one.

### NLRP3 alkylation leading to the inhibition of its ATPase activity is relevant to the molecular action of BOT-4-one

**Figure 5:**
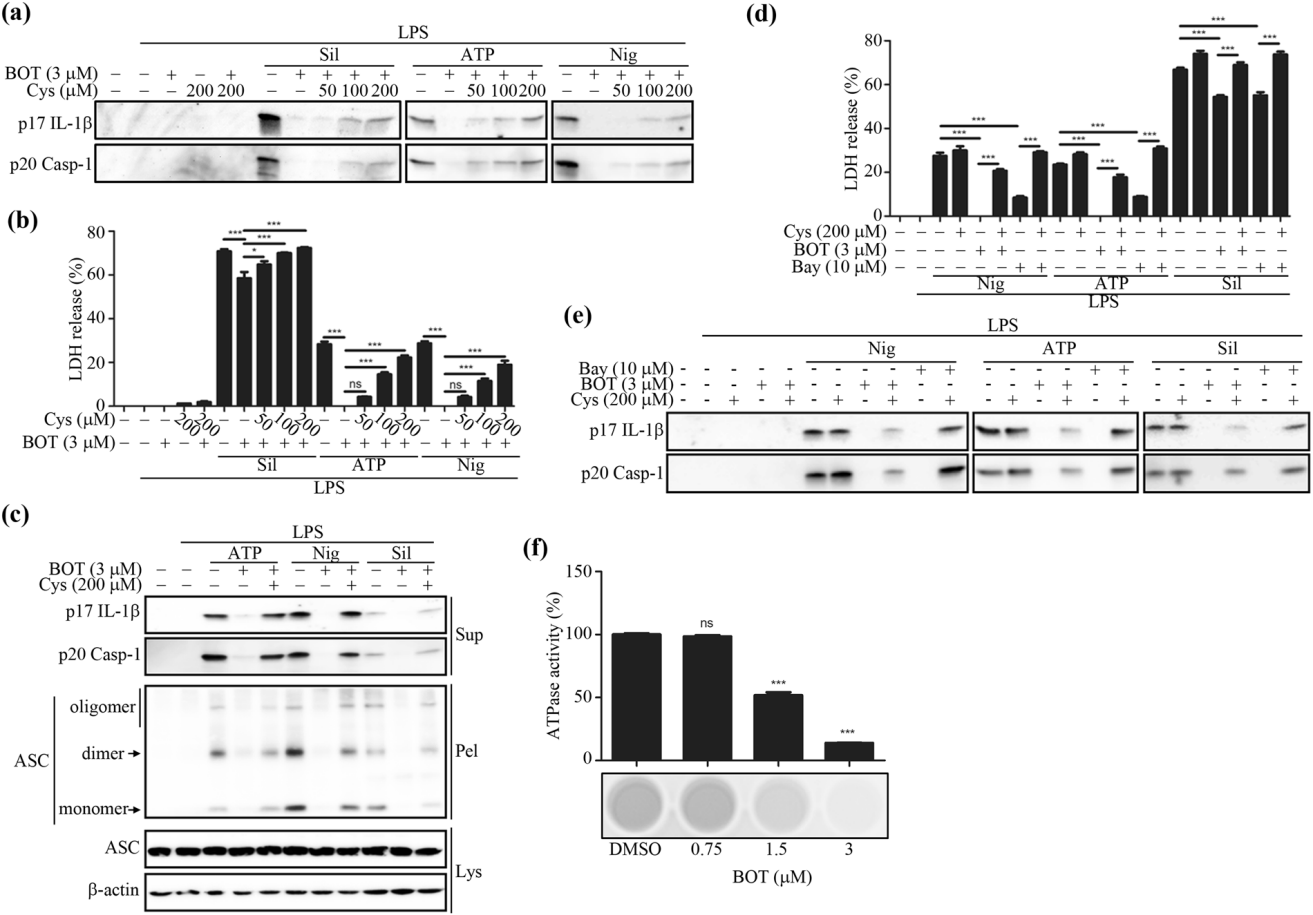
Involvement of BOT-4-one on the the inhibition of the ATPase activity of NLRP3 and the inhibition of NLRP3 inflammasome activation through NLRP3 alkylation. LPS-primed BMDMs were treated with BOT-4-one or Bay11-7082 (Bay) for 1 h in the presence or absence of L-cysteine (Cys) and then stimulated with nigericin (Nig), ATP, or silica crystals (Sil). (**a** and **e**) The supernatants were analyzed by immunoblot. Full-length blots/gels are presented in Supplementary Fig. S12a and b. (**b** and **d**) The LDH release in the cell supernatants was measured by LDH assay. (**c**) ASC oligomerizations in cross-linked cytosolic pellets were analyzed by immunoblot. Full-length blots/gels are presented in Supplementary Fig. 12c. (**f**) The amount of NLRP3-mediated ATP converted into ADP in the presence or absence of BOT-4-one was determined by luminescence by using the ADP-Glo assay. The data represent the mean ± SEM of three independent experiments; *p < 0.05; ***p < 0.001; ns: non-significant.

BOT-4-one has been previously identified as an immunomodulatory agent that inhibits the NF-κB pathway by IKKβ alkylation^17^, indicating that BOT-4-one has the capability of protein alkylation. We confirmed that this alkylating activity of BOT-4-one can be efficiently inhibited by the alkylation inhibitor L-cystein; i.e., the phosphorylation and degradation of IκB-α inhibited by BOT-4-one was restored in the presence of L-cysteine (Supplementary Fig. S5). Meanwhile, a known NLRP3 alkylator, Bay11-7082, has also been identified as an NF-κB inhibitor inducing Ubc (ubiquitin conjugating) 13 and UBCH7 alkylation^9,34^. We thus examined first whether the BOT-4-one effects on the NLRP3 inflammasome pathways are also associated with its alkylating activity. The results showed that all of the observed NLRP3 inflammasome-mediated signals (IL-1β and caspase-1 maturation, LDH release, and ASC insolubilization) suppressed by BOT-4-one could be restored in the presence of the alkylation inhibitor L-cysteine (Fig. 5a–c). Likewise, the NLRP3-inhibitory functionality of Bay11-7082 could also be blocked by L-cysteine (Fig. 5d and e). Collectively, the results suggested that BOT-4-one, like Bay11-7082, probably acts as an efficient NLRP3 alkylator, as well as functioning as an NF-κB pathway inhibitor.

Generally, a primary consequence of NLRP3 alkylation by other known specific inhibitors of the NLRP3 inflammasome, including Bay11-7082, MNS, and some acrylamide derivatives, is appreciated as the attenuation of the ATPase activity of NLRP3^9–11^. In this regard, the effect of BOT-4-one on the *in vitro* ATPase activity of recombinant NLRP3 was assessed (Fig. 5f). The result finally demonstrated that the alkylation-induced attenuation of ATPase activity could be relevant to the molecular action of BOT-4-one on NLRP3.

### NLRP3 alkylation enhances NLRP3 ubiquitination and hinders inflammasome assembly

**Figure 6:**
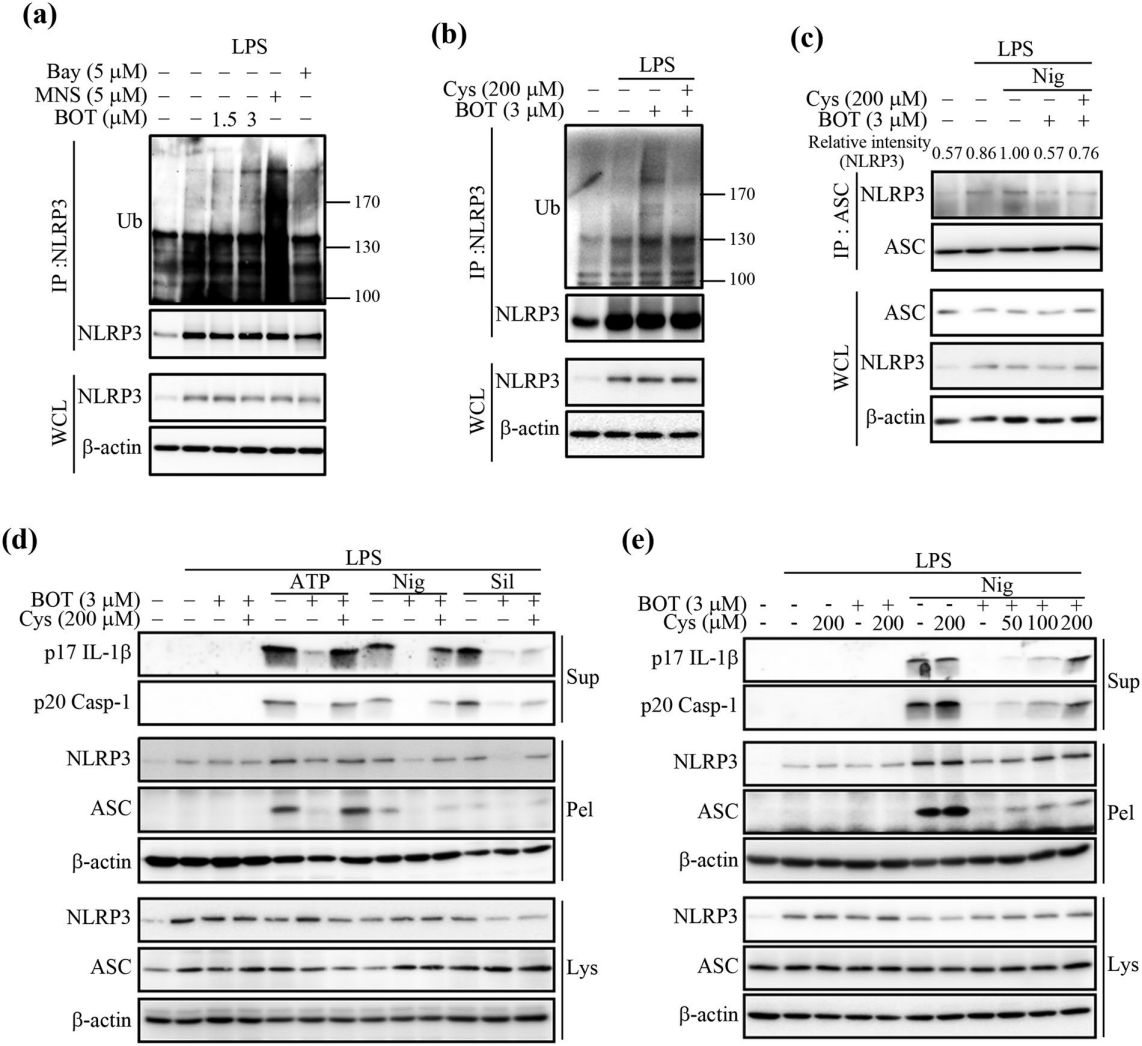
Alkylation of NLRP3 by BOT-4-one promotes the ubiquitination of NLRP3 and attenuates NLRP3 inflammasome assembly. (**a**) LPS-primed BMDMs were treated with BOT-4-one, MNS, or Bay11-7082 (Bay) for 1 h. (**b**) LPS-primed BMDMs were treated with L-cysteine (Cys) for 15 min and then treated with BOT-4-one for 1 h. (**a** and **b**) NLRP3 immunoprecipitates were analyzed for ubiquitination. Full-length blots/gels are presented in Supplementary Fig. S13a and b. (**c**) LPS-primed BMDMs were treated with BOT-4-one for 1 h in the presence or absence of L-cysteine (Cys) and then stimulated with nigericin (Nig). The NLRP3/ASC interaction was analyzed by immunoprecipitation and immunoblot. Full-length blots/gels are presented in Supplementary Fig. S13c. (**d** and **e**) LPS-primed BMDMs were treated with BOT-4-one for 1 h in the presence or absence of the indicated concentrations of L-cysteine (Cys) and then stimulated with ATP, nigericin (Nig), or silica crystals (Sil). The supernatants from the cell cultures (Sup), soluble lysates (Lys), and insoluble pellets (Pel) of Triton × 100 were analyzed by immunoblot. Full-length blots/gels are presented in Supplementary Fig. S13d and e.

In addition to ATPase activity, the deubiquitination process of NLRP3 is known to be involved in NLRP3 inflammasome activation^35,36^. It has also recently been suggested that both ATPase activity and the ubiquitination of NLRP3 are regulated by PKA^8,37^. We first made sure that the BOT-4-one action would not be influenced by PKA activation by observing that the PKA inhibitor H-89 could not prevent the BOT-4-one-mediated inhibition of NLRP3 inflammasome activation, whereas it restored the NLRP3 inflammasome functionality (maturation of IL-1β and caspase-1) suppressed by the adenylyl cyclase activator forskolin (Supplementary Fig. S6). Then, we examined whether BOT-4-one-induced NLRP3 alkylation could also affect the ubiquitination of NLRP3. Notably, an appreciable increase of NLRP3 ubiquitination was observed in the presence of BOT-4-one (Fig. 6a and b). Although it cannot be distinguished whether BOT-4-one directly promoted NLRP3 ubiquitination or inhibited the deubiquitination process of NLRP3, this PKA-independent enhancement of NLRP3 ubiquitination could be due to NLRP3 alkylation, as it was also induced by other NLRP3 alkylators, Bay11-7082 and MNS (Fig. 6a), and efficiently blocked by L-cysteine (Fig. 6b). NLRP3 ubiquitination is generally expected to obstruct the proper interaction between NLRP3 and ASC that is important for their co-localization into insoluble fractions^37^. Supporting this assumption, the immunoprecipitation result showed a reduced assembly of NLRP3 and ASC in the presence of BOT-4-one, which can be prevented by pre-treatment with L-cysteine (Fig. 6c). Consequently, the Triton X-100-insoluble NLRP3 and ASC levels that were reduced by BOT-4-one could also be restored in the presence of L-cysteine in both mouse (Fig. 6d and e) and human cells (Supplementary Fig. S7). These observations suggest that the attenuation of the NLRP3 inflammasome could be partly attributable to the enhanced level of NLRP3 ubiquitination, as well as the impaired ATPase activity, by the BOT-4-one-mediated NLRP3 alkylation.

### BOT-4-one exhibits an *in vivo* anti-inflammatory activity on MSU-induced peritonitis in mice

**Figure 7:**
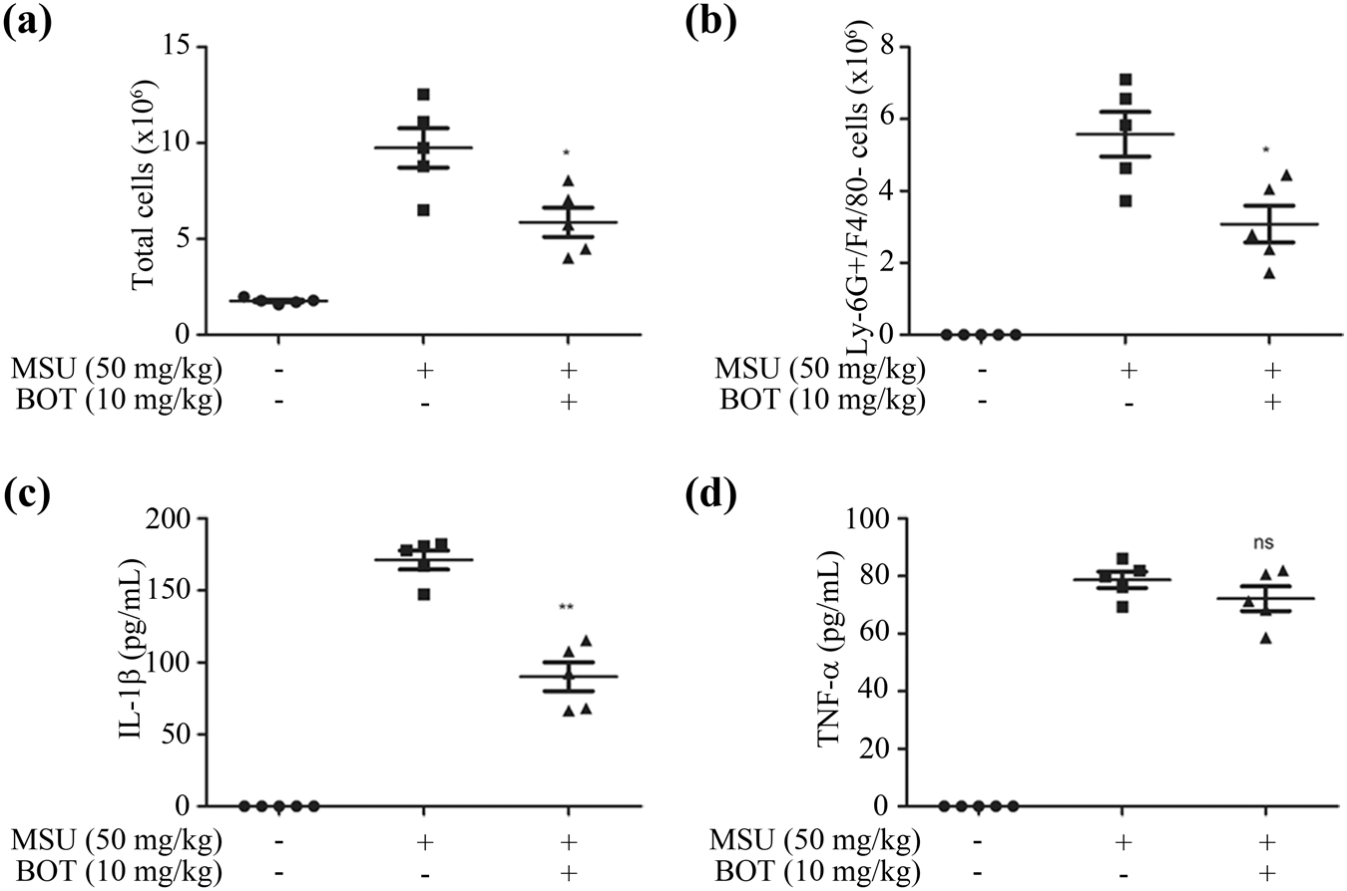
BOT-4-one suppresses MSU-induced peritonitis *in vivo* model. C57BL/6 mice were given an intraperitoneal injection of BOT-4-one successively 2 and 12 h before infection with MSU. (**a**) Peritoneal lavage fluids were collected at 6 h after the MSU challenge to measure the total number of cells. (**b**) Ly-6G^+^/F4/80^−^ cells were measured by flow cytometry. Concentrations of IL-1β (**c**) and TNF-α (**d**) in the peritoneal lavage fluids were measured with ELISA. The data represent the mean ± SEM of three independent experiments; *p < 0.05; **p < 0.01; ns: non-significant.

Based on the potent inhibition of NLRP3 inflammasome activation, we assessed the utility of BOT-4-one as an anti-inflammatory agent by using a peritonitis mouse model. The peritonitis was induced by monosodium urate (MSU), which is a well-known activator of the NLRP3 inflammasome and widely used for the NLRP3 inflammasome-related *in vivo* disease model^38,39^. Compared to the non-treated group, the MSU-administered mice showed an increased total number of peritoneal cells (Fig. 7a) that particularly included a large amount of Ly-6G^+^/F4/80^−^ neutrophils (Fig. 7b), indicative of an acute peritonitis symptom. In contrast, the administration of BOT-4-one significantly decreased the MSU-mediated recruitment of Ly-6G^+^/F4/80^−^ neutrophils and reduced the total number of peritoneal cells. Finally, an appreciable suppression of the MSU-induced IL-1β production in peritoneal lavage fluid was observed by the BOT-4-one administration (Fig. 7c), although the inhibition of TNF-α production was not apparent (Fig. 7d). Collectively, these data indicated that BOT-4-one has a useful protective effect on MSU-induced peritonitis by inhibiting the NLRP3 inflammasome-mediated IL-1β production.

## Discussion

We demonstrated that BOT-4-one exhibited a potent inhibition of the NLRP3 inflammasome in both mouse and human cells and in a peritonitis mouse model. Other known chemicals, including Bay11-7082, MNS, and acrylamide derivatives, which also selectively inhibit the activation of the NLRP3 inflammasome, have been suggested as probably acting through a direct alkylation of NLRP3^9–11^. Although such a covalent binding has not been elucidated yet with direct experimental evidences, such as crystal structure and mass spectrometry, cysteine residues were considered as their alkylating target sites through computational and modeling analysis^11^. Generally, cysteine is frequently involved in the modulation of protein activity and signaling events through the potent chemical reactivity at its thiol group^40^. In particular, the cysteine thiol group is easily alkylated (referred to as S-alkylation) by some electrophilic chemicals or peptides^41^, whereas this alkylation can be experimentally inhibited by L-cysteine^9,10^. A previous report showed that BOT-4-one also has a potentially electrophilic site that can be directly linked to the Cys179 residue of IKKβ by a possible chemical reaction of nucleophilic addition^17^. In the present study, BOT-4-one treatment resulted in an impaired ATPase activity of NLRP3, which is known as a primary resultant of NLRP3 alkylation by other known NLRP3 alkylators^9–11^. In addition, all of the BOT-4-one functionality could be suppressed by L-cysteine and the inactivation by L-cysteine was also relevant to the known NLRP3 alkylators. These observations suggest that inducing the direct alkylation of NLRP3 could also be relevant to the BOT-4-one action for inhibiting the activation of the NLRP3 inflammasome, although the exact alkylation site in NLRP3 remains to be identified.

Although BOT-4-one did not affect the activation of the AIM2 inflammasome, a slight inhibitory effect of BOT-4-one on the NLRC4 inflammasome was detectable just on the IL-1β and caspase-1 maturation. Such a partial inhibitory effect on NLRC4 inflammasome activation has also been observed for the action of Bay11-7082^9^. As discussed by the authors, various possibilities can be raised for this marginal inhibition of NLRC4, including a direct, albeit marginal, alkylation of NLRC4. In addition, the partial inhibition of the NLRC4 inflammasome by BOT-4-one could also be prevented in the presence of L-cysteine (Supplementary Fig. S8), implying that it was related to the alkylating capability of BOT-4-one. However, a recent study showed that NLRP3 contributes to NLRC4 inflammasome activation and the activated NLRC4 inflammasome also recruits NLRP3 and ASC^23^. Therefore, an alternative possibility cannot be excluded that preventing NLRP3 inflammasome activation by BOT-4-one interfered with the function of the activated NLRC4 inflammasome; i.e., the NLRP3 alkylation might also be responsible for the marginal suppression of the outcomes of NLRC4 inflammasome signals, as observed in the NLRP3-deficient macrophages^23^.

NLRP3 alkylation is generally expected to reduce its ATP-binding affinity^9^, thereby resulting in impaired ATPase activity^10,11^. It has been previously shown that an NLRP3 mutant with impaired ATPase activity failed in self-oligomerization and the subsequent translocation into the detergent-insoluble fraction^7^, which are important processes of inflammasome activation^26–28,31^. The ATPase activity of NLRP3 is also important for its oligomeric association with ASC^7,8,10^. In the present study, we proved the reduced *in vitro* ATPase activity of recombinant NLRP3 protein in the presence of BOT-4-one. In addition, the results showing a weakened association between NLRP3 and ASC oligomers and the concomitant reduction in their distribution into the insoluble fraction corroborate that BOT-4-one action in cells could indeed be displayed by the alkylation-mediated inhibition of the ATPase activity of NLRP3.

As well as the ATPase activity, the deubiquitination of NLRP3 is required for the NLRP3 inflammasome assembly and its subsequent activation^35–37^. We showed in this investigation that the ubiquitination level of NLRP3 was up-regulated by BOT-4-one. Although blocking NLRP3 deubiquitination has been reported to result in the complete inhibition of NLRP3 inflammasome activation^35,36^, determination cannot be made regarding whether the increased level of NLRP3 ubiquitination in the presence of BOT-4-one was due to the suppression of deubiquitination or the promotion of ubiquitination. However, it can be reasonably regarded that the observed enhancement of ubiquitination was attributed to the NLRP3 alkylation, as other known NLRP3 alkylators showed the same functionality and can be commonly inhibited by L-cysteine. Such a dual effect with decreased ATPase activity and increased ubiquitination of NLRP3 has been recently observed by PKA activation^8,37^, but exact correlations between the two processes have not been elucidated^8,37^. Likewise, it remains to be determined whether the alkylation-mediated enhancement of NLRP3 ubiquitination, which is PKA-independent, is attributed to the reduced ATPase activity or to the alkylation functions as another independent signal for ubiquitination. However, the present study constitutes the first experimental evidence indicating that the alkylation of NLRP3 is involved in the regulation of its ubiquitination level.

The ATPase activity of NLRP3 is appreciated as a useful target for the development of NLRP3 inflammasome inhibitors^12^. The present study revealed that BOT-4-one can effectively target NLRP3 ATPase activity, thereby exhibiting a potent inhibitory activity on the NLRP3 inflammasome, with an estimated submicromolar IC_50_ value. BOT-4-one can be evaluated as superior to other known NLRP3 inflammasome-specific inhibitors, including glyburide^42^, Bay11-7082^9^, and MNS^10^, which exhibit micromolar IC_50_ values. Meanwhile, MCC950^26^, a recently developed NLRP3 inflammasome inhibitor with nanomolar IC_50_, has not been clearly validated in its mode of action. Although, BOT-4-one can alkylate other proteins such as IKKβ or NLRC4, the inhibitory capacity of BOT-4-one on these proteins is mild when compared to NLRP3. Furthermore, IKKβ is completely inhibited with 30 μM of BOT-4-one which is 10 fold higher than required concentration for NLRP3 inhibition^17^. Therefore, the potency of BOT-4-one is superior to NLRP3 than IKKβ or NLRC4. It remains to be eulcidated as to which protein between NLRP3 and IKKβ is specific to BOT-4-one. Taken all together, we expect that BOT-4-one will not only provide a useful tool for exploring the NLRP3 inflammasome but also contribute to the development of a valuable therapeutic strategy in treating various NLRP3 inflammasome-related auto-immune and inflammatory diseases.

## Materials and Methods

### Reagents and antibodies

BOT-4-one was provided by Sang-Kyu Ye and Byung-Hak Kim (Seoul National University of College of Medicine, South Korea)^17^. Penicillin-streptomycin, fetal bovine serum (FBS), opti-MEM, and RPMI 1640 were purchased from Gibco (Grand Island, NY, USA). IL-1β antibody (AF401)^8^ and IL-1β enzyme-linked immunosorbent assay (ELISA) kit were purchased from R&D Systems (Minneapolis, MN, USA). Antibodies against ASC (AL177)^8^, NLRP3 (Cryo-2)^8^, and caspase-1 (clone Casper-1)^8^ were purchased from Adipogen (San Diego, CA, USA). β-actin (sc-1616)^43^ and ubiquitin (sc-8017)^37^ were purchased from Santa Cruz Biotechnology (Santa Cruz, CA, USA). Monosodium urate (MSU) crystal, Bay11-7082, silica crystal, nigericin, flagellin from *S. typhimurium*, poly(dAdT), Pam3CSK4, and zVAD-FMK were purchased from InvivoGen (San Diego, CA, USA). Mouse TNF-α ELISA Ready-SET-Go was purchased from eBioscience (San Diego, CA, USA). A western blot chemiluminescence reagent kit was purchased from Pierce Chemical (Rockford, IL, USA). Polyvinylidene fluoride (PVDF) and nitrocellulose membrane were purchased from Millipore Corporation (Bedford, MA, USA). LPS (*Escherichia. coli* 026:B6 and 011:B4), 3,4-methylenedioxy-β-nitrostyrene, and ATP were purchased from Sigma-Aldrich (St.Louis, MO, USA). All other chemicals used were of the highest quality among those that are commercially available.

### Animals

Female C57BL/6 mice (weighing 22–25 g, six weeks old) were purchased from SAMTAKO Co., Korea. The animals were housed in groups of five under standard conditions (temperature 22  ± 2 °C, humidity 55 ± 5%, 12/12-h light/dark cycle) with food and water *ad libitum*. All experiments were performed under the guidelines of the Konkuk University Animal Care Committee. The study protocol was reviewed and approved by the Ethics of Animal Experiments Committee of Konkuk University (Republic of Korea) (Permit No. KU16193).

### Cell culture and stimulation

Bone-marrow derived macrophages (BMDMs) were collected and cultured as described^43^. BMDMs were primed with LPS (100 ng/mL) for 4 h. After LPS priming, the medium was replaced with Opti-MEM, and cells were incubated for 1 h with or without BOT-4-one, KCl (150 mM), or zVAD (20 μM) before being stimulated for 1 h with ATP (5 mM) or nigericin (10 μM), for 3 h with silica crystals (150 μg/mL) and transfected for 1 h with poly(dA:dT) (2 μg/mL) or for 3 h with flagellin (1.5 μg/mL) by using Lipofectamine 2000 (Invitrogen).

For noncanonical inflammasome activation, cells were primed with Pam3CSK4 (200 ng/mL) for 3 h. After priming, the medium was replaced with Opti-MEM and cells were incubated for 1 h with or without BOT-4-one, or KCl (150 mM) before transfection with LPS (*E.coli* 011:B4) (70 μg/mL) for 3 h using Lipofectamine 2000 (Invitrogen).

### ASC oligomer cross-linking and ASC speck staining

For ASC oligomer cross-linking, cells were lysed in an A0 buffer (0.5% Triton × 100, 20 mM HEPES-KOH, pH 7.5, 150 mM KCl, and complete protease and phosphatase inhibitor cocktail) on ice by syringing 10 times through a G26 needle. The cell lysates were centrifuged at 6000 rpm at 4 °C for 10 min. Pellets were resuspended in PBS and crosslinked with disuccinimidyl suberate (DSS) (2 mM) (Thermo Scientific-Pierce, Rockford, IL, USA). The cross-linked pellets were centrifuged at 13000 rpm for 15 min and dissolved directly in a SDS sample buffer^29^.

For ASC speck staining, inflammasome activated cells were fixed with 4% paraformaldehyde, permeabilized with acetone, and blocked with 10% horse serum. Cells were stained with ASC antibody (sc-22514-R, Santa Cruz)^44^ and Cy3-conjugated anti-rabbit antibody (711-165-152, Jackson ImmunoResearch Lab)^45^. Nuclei were counterstained with DAPI (Sigma-Aldrich)^46^. All of the images were captured with a fluorescence microscope (Axio, Carl Zeiss).

### Separation of soluble and insoluble fractions of Triton × 100 from cell lysates

Cells were lysed with TTNE (1% Triton × 100, 50 mM Tris-HCl, pH 7.4, 150 mM NaCl, 2 mM EDTA, and complete protease and phosphatase inhibitor cocktail). The lysates were centrifuged at 6000 rpm at 4 °C for 15 min and the supernatants and pellets were used as the soluble and insoluble fractions of Triton × 100, respectively^24^.

### Measurement of ATPase activity of NLRP3

The ATPase activity of NLRP3 was measured as described previously^11^. Human recombinant NLRP3 (BPS Bioscience, San Diego, CA, USA) was incubated with DMSO or BOT-4-one in reaction buffer (20 mM Tris HCl, pH 7.8, 133 mM NaCl, 20 mM MgCl_2_, 3 mM KCl, and 0.56 mM EDTA) for 20 min at 37 °C. ATP (250 mM, ultra-pure ATP) was added, and the mixtures were further incubated for 40 min at 37 °C. The hydrolysis of ATP by NLRP3 was determined by an ADP-Glo Kinase Assay (Promega, Madison, WI, USA) according to the manufacturers’ protocol^11^.

### Immunoprecipitation

To detect NLRP3/ASC interaction, cells were lysed with IP buffer (1% NP-40, 50 mM Tris-HCl, pH 7.4, 150 mM NaCl, 0.5% sodium deoxycholate, and complete protease and phosphatase inhibitor cocktail) and sonicated for 5 sec at 20 amp. The lysates were precleared and incubated with ASC antibody (sc-22514-R, Santa Cruz)^46^ overnight at 4 °C^46^.

To detect NLRP3 ubiquitination, BMDMs were lysed with TTNE. The lysates were precleared and incubated with NLRP3 antibody overnight at 4 °C. the beads were rinsed with lysis buffer and eluted (0.1 M Tris-HCl, pH 6.8, 0.4% sodium dodecyl sulfate, 12% glycerol, 0.286 M β-mercaptoethanol, and 0.32% bromophenol blue)^35^. The lysates were precleared and incubated with NLRP3 antibody (Cryo-2, Adipogen)^35^ overnight at 4 °C.

### MSU-induced mouse peritonitis

BOT-4-one (10 mg/kg) was administered intraperitoneally (i.p.) before 2 and 12 h of i.p. injection of MSU (50 mg/kg) or PBS. Six hours after the MSU challenge, all of the animals were sacrificed. Peritoneal cells were obtained with 10 mL of PBS. The recovered fluid was pelleted by centrifugation, and the total cells were counted. Subsequently, the cells were stained for both APC-conjugated F4/80 (17-4801) and FITC-conjugated Ly-6G (11-5931) antibodies (eBioscience)^47^ and analyzed using flow cytometry (BD San Diego, CA, USA). The number of neutrophils was calculated as total cells multiplied by the percentage of Ly-6G positive and F4/80 negative cells. The concentrations of IL-1β and TNF-α in the supernatants of the peritoneal lavage fluid were determined by ELISA.

### Statistical analysis

The results are expressed as the mean ± standard error of the mean (SEM) of at least three independent experiments (n = 3). Statistical analysis was performed using the Student’s *t*-test with GraphPad (San Diego, CA, USA) prism software, and *p*-values less than 0.05 were considered to be statistically significant.

## Electronic supplementary material

Supplementary information
