# Supplementary material for: BOT-4-one attenuates NLRP3 inflammasome activation: NLRP3 alkylation leading to the regulation of its ATPase activity and ubiquitination
Source: Sci Rep. 2017 Nov 8;7:15020. doi: 10.1038/s41598-017-15314-8 (PMC5678161; doi:10.1038/s41598-017-15314-8)
Supplement: Supplementary file 1 — Supplementary information [file 41598_2017_15314_MOESM1_ESM.pdf]

Supplementary Materials for

BOT-4-one attenuates NLRP3 inflammasome activation: NLRP3 alkylation leading to the regulation of its ATPase activity and ubiquitination

Do-Wan Shim[a], Woo-Young Shin[a], Sang-Hyeun Yu[a], Byung-Hak Kim[b,c] Sang-Kyu Ye[b], Sushruta Koppula[d], Hyung-Sik Won[a,d], Tae-Bong Kang[a,d,]* and Kwang-Ho Lee[a,d,]*

This PDF file includes:

Figs. S1 to S13

Figure legends for Figs. S1 to S8

Supplementary Materials and Methods

Supplementary Figures and Captions

SFig. 1

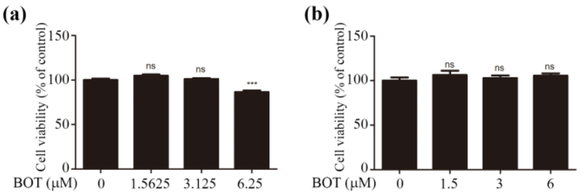

SFig. 1. Effect of BOT-4-one on cell viability of BMDMs or PMA-differentiated THP-1 cells. BMDMs (**a**) or PMA-differentiated THP-1 cells (**b**) were treated with various concentrations of BOT-4-one. Cell viability was measured by MTT assay. Data represent the mean ± SEM of three independent experiments; ***$p < 0.001$; ns: non-significant.

SFig. 2

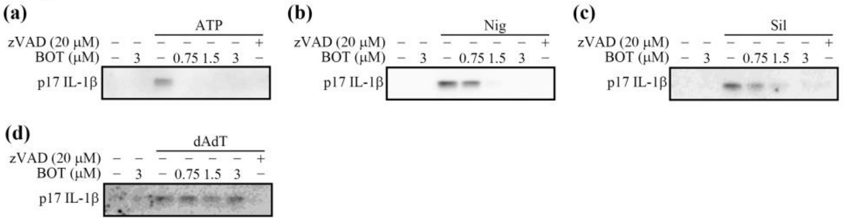

SFig. 2. BOT-4-one inhibits NLRP3 inflammasome activation in PMA-differentiated THP-1 cells. (**a-d**) PMA-differentiated THP-1 cells were treated with different concentrations of BOT-4-one or zVAD for 1 h, and then stimulated with ATP, nigericin (Nig), silica crystals (Sil), or poly(dA:dT) (dAdT). Supernatants were analyzed by immunoblot analysis.

SFig. 3

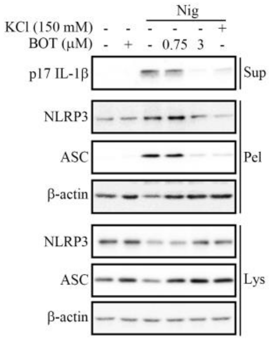

SFig. 3. BOT-4-one inhibits NLRP3-dependent insolubilization of both NLRP3 and ASC in PMA-differentiated THP-1 cells. PMA-differentiated THP-1 cells were treated with BOT-4-one or KCl for 1 h, and then stimulated with nigericin (Nig) for 1 h. Cell culture supernatants (Sup), Triton X 100 soluble lysates (Lys) and insoluble pellets (Pel) were analyzed by immunoblot analysis.

Sfig. 4

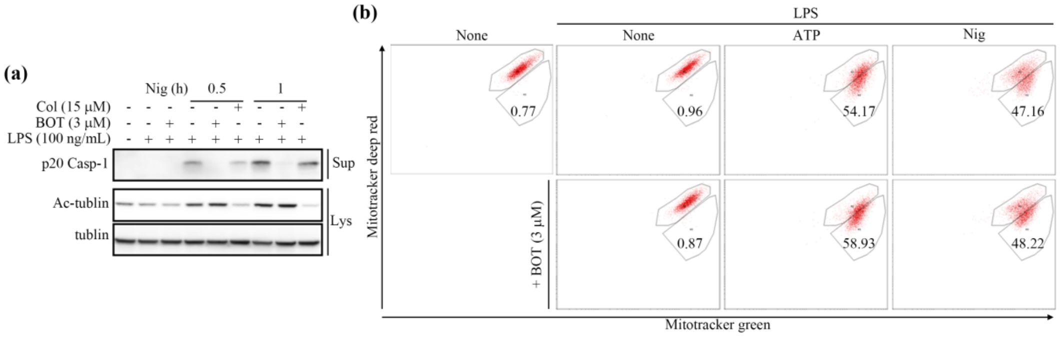

SFig. 4. BOT-4-one does not affect tubulin acetylation and mitochondrial membrane potential. (**a**) LPS-primed BMDMs were treated with BOT-4-one or colchicine (Col) for 1 h, and then stimulated with nigericin (Nig) for 1 h. Supernatants (Sup) and cell lysate (Lys) were analyzed by immunoblot analysis. (**b**) LPS-primed BMDMs were treated with BOT-4-one for 1 h followed by stimulation with ATP or nigericin (Nig) for 1 h, and then stained with Mitotracker Green and Mitotracker Deep Red.

SFig. 5

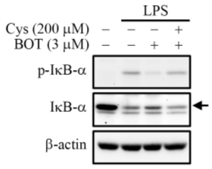

SFig. 5. BOT inhibits LPS-mediated NF-κB activation through IKKβ alkylation in BMDMs. BMDMs were treated with BOT-4-one for 1 h in the presence or absence of L-cysteine (Cys), and then stimulated with LPS for 30 min. Cell lysates were analyzed by immunoblotting with indicated Abs.

SFig. 6

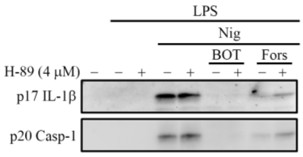

SFig. 6. BOT-4-one inhibits NLRP3 inflammasome activation in a PKA-independent manner. LPS-primed BMDMs were treated with BOT-4-one (BOT, 3 μM) or forskolin (Fors, 50 μM) for 1h in the presence or absence of H-89, and then stimulated with nigericin (Nig). The supernatants were analyzed by immunoblot.

SFig. 7

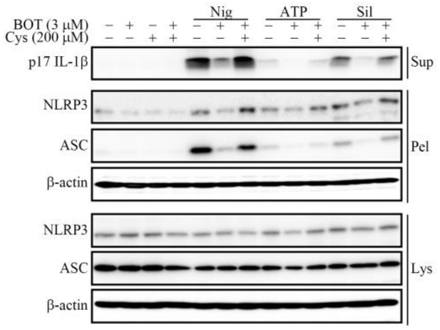

SFig. 7. BOT-4-one inhibits insolubilization of both NLRP3 and ASC through induction of NLRP3 alkylation in THP-1 cells. PMA-differentiated THP-1 cells were treated with BOT-4-one for 1 h in the presence or absence of L-cysteine (Cys), and then stimulated with nigericin (Nig), ATP, or silica crystals (Sil). Cell cultured supernatants, Triton X 100 soluble lysates (Lys), and insoluble pellets (Pel) were analyzed by immunoblot analysis.

SFig. 8

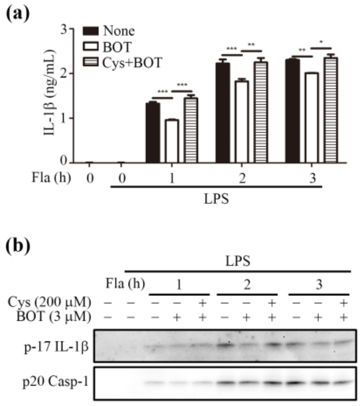

SFig. 8. BOT-4-one slightly inhibits NLRC4 inflammasome activation through alkylating activity of BOT-4-one. LPS-primed BMDMs were treated with BOT-4-one for 1 h in the presence or absence of L-cysteine (Cys), and then stimulated with flagellin (Fla). (**a**) IL-1β secretion in the cell supernatants was measured by ELISA. (**b**) Cell cultured supernatants (Sup) were analyzed by immunoblot analysis. $*p < 0.05$; $**p < 0.01$; ***p < 0.001.

SFig. 9

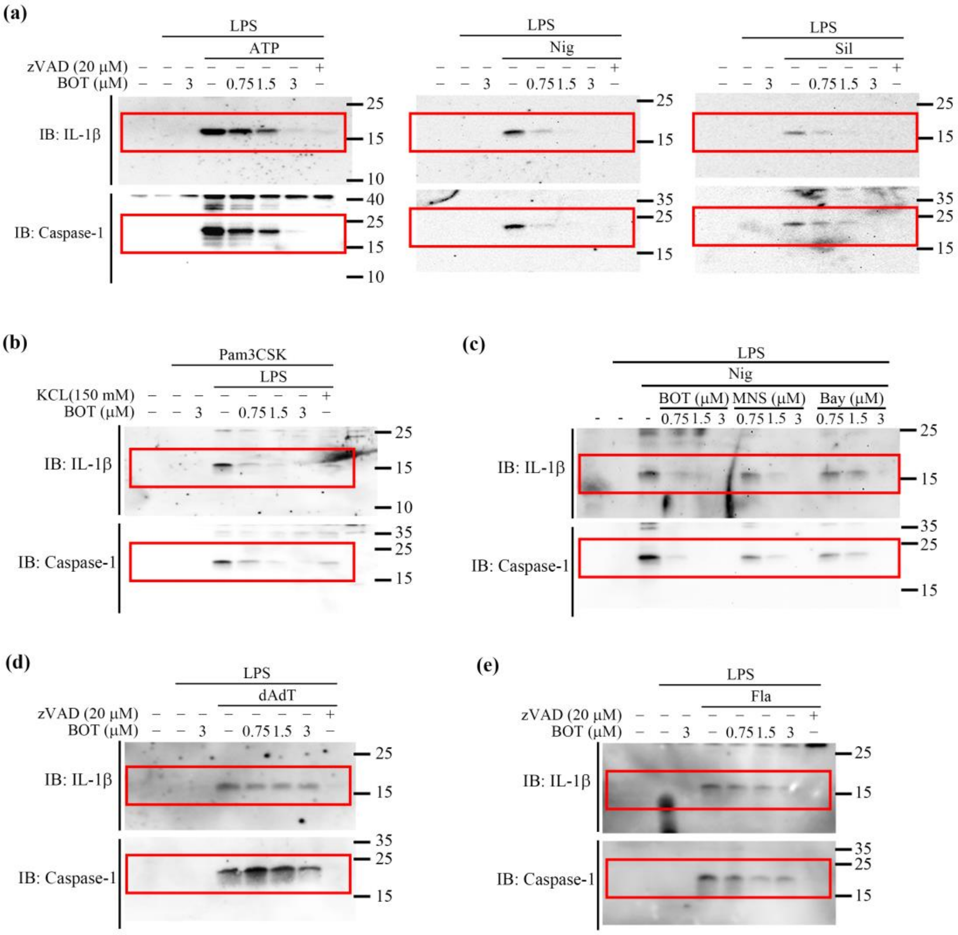
(a)
LPS
ATP
zVAD (20 μM)
BOT (μM)
IB: IL-1β
IB: Caspase-1
Nig
Sil
(b)
Pam3CSK
LPS
KCL(150 mM)
(c)
BOT (μM)
MNS (μM)
Bay (μM)
(d)
dAdT
(e)
Fla

SFig. 10

(a)

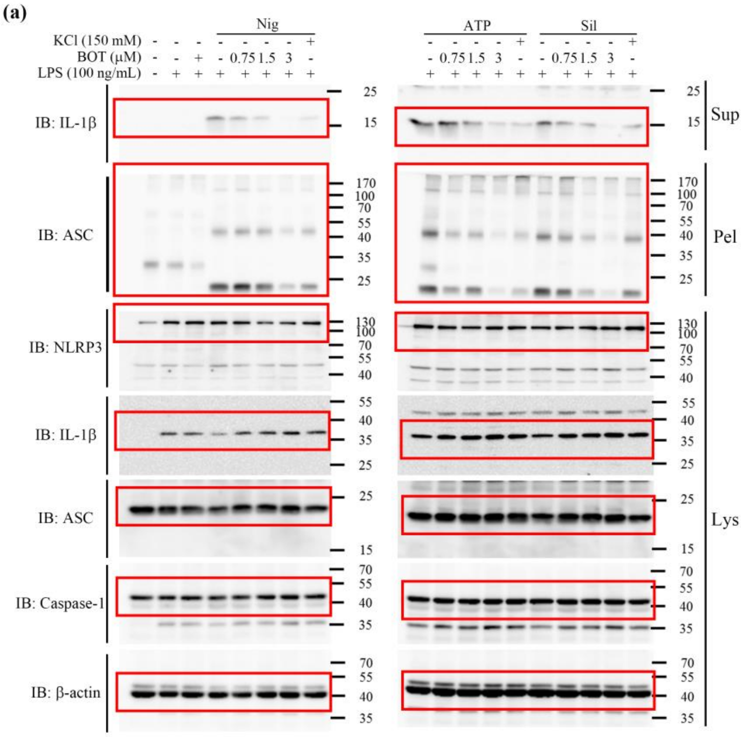

Nig
ATP
Sil
KCl (150 mM)
BOT (μM)
LPS (100 ng/mL)
IB: IL-1β
IB: ASC
IB: NLRP3
IB: IL-1β
IB: ASC
IB: Caspase-1
IB: β-actin
Sup
Pel
Lys

(b)

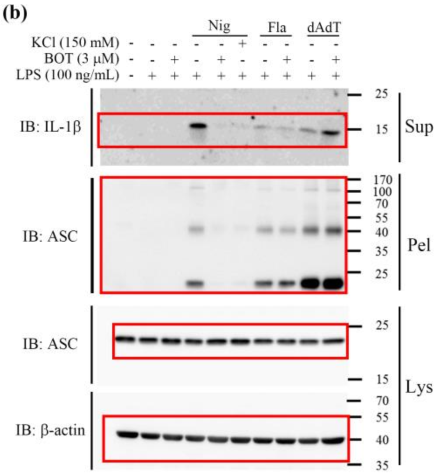

Nig
Fla
dAdT
KCl (150 mM)
BOT (3 μM)
LPS (100 ng/mL)
IB: IL-1β
IB: ASC
IB: ASC
IB: β-actin
Sup
Pel
Lys

SFig. 11

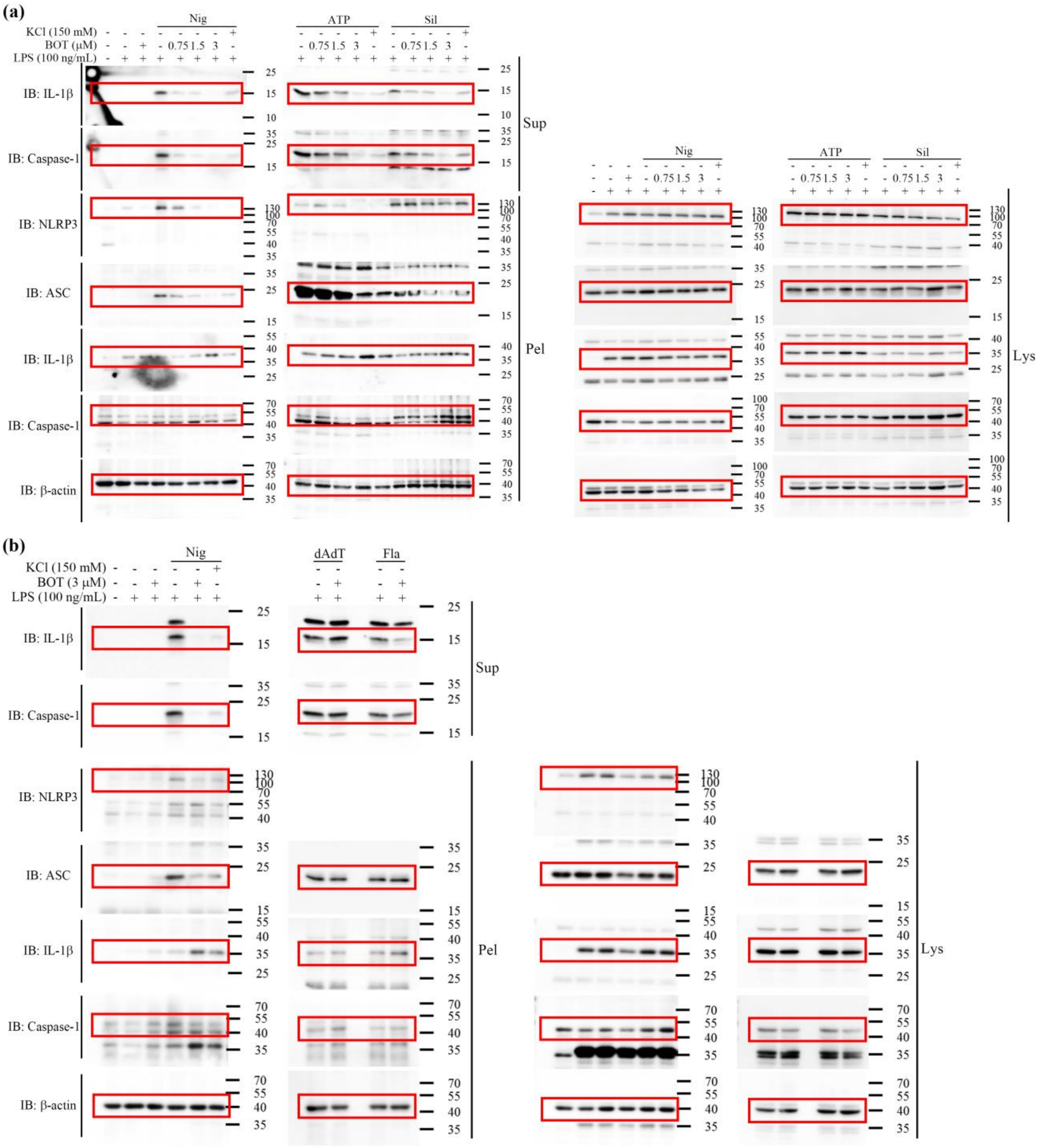
(a)
KCl (150 mM)
BOT (μM)
LPS (100 ng/mL)
Nig
ATP
Sil
IB: IL-1β
IB: Caspase-1
IB: NLRP3
IB: ASC
IB: β-actin
Sup
Pel
Lys
(b)
BOT (3 μM)
dAdT
Fla

SFig. 12

(a)

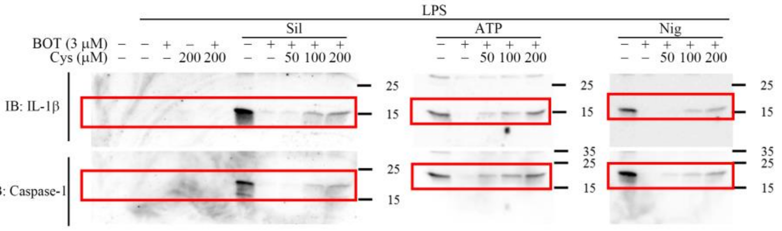
LPS
Sil
ATP
Nig
BOT (3 μM)
Cys (μM)
IB: IL-1β
IB: Caspase-1
25
15
35

(b)

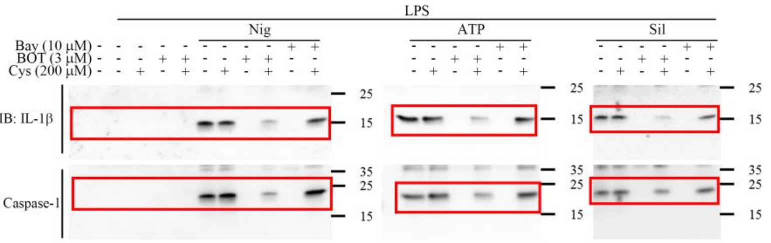
LPS
Nig
ATP
Sil
Bay (10 μM)
BOT (3 μM)
Cys (200 μM)
IB: IL-1β
IB: Caspase-1
25
15
35

(c)

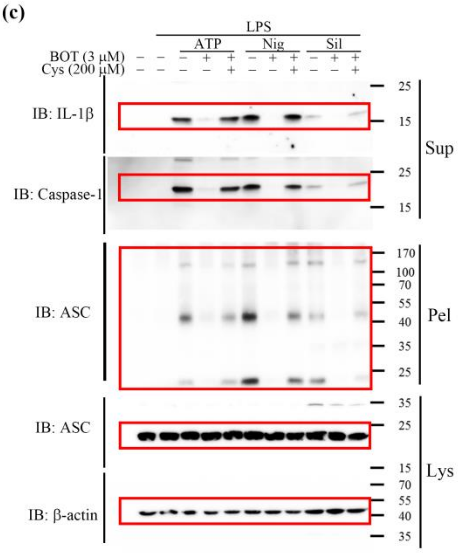
LPS
ATP
Nig
Sil
BOT (3 μM)
Cys (200 μM)
IB: IL-1β
IB: Caspase-1
IB: ASC
IB: ASC
IB: β-actin
Sup
Pel
Lys
170
100
70
55
40
35
25
15

SFig. 13

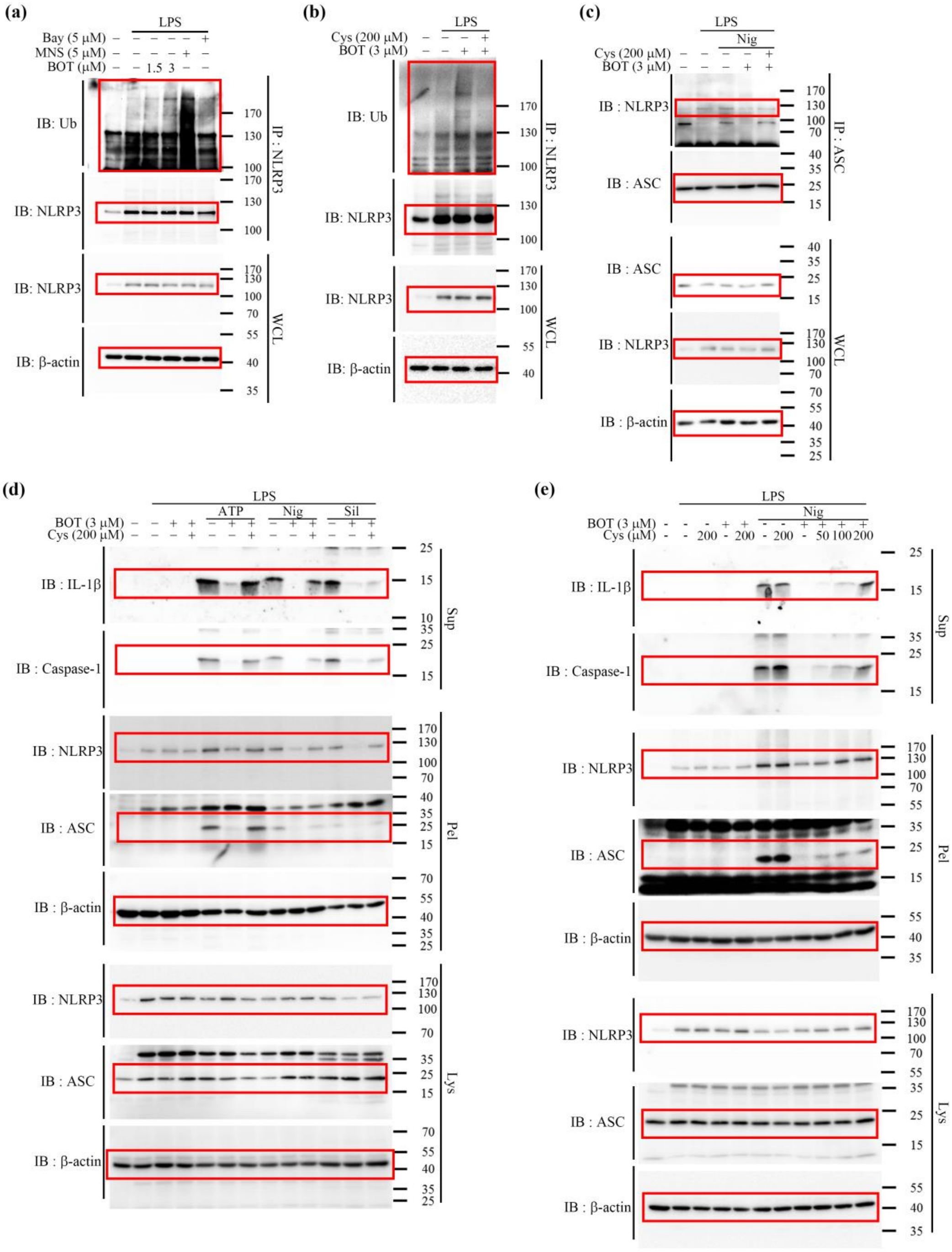

(a)
LPS
Bay (5 μM)
MNS (5 μM)
BOT (μM)
IB: Ub
IB: NLRP3
IB: β-actin
IP : NLRP3
WCL
(b)
Cys (200 μM)
BOT (3 μM)
(c)
Nig
IB : NLRP3
IB : ASC
IB : β-actin
IP : ASC
(d)
ATP
Sil
IB : IL-1β
IB : Caspase-1
Sup
Pel
Lys
(e)
Cys (μM)

Supplementary Materials and Methods

Cell culture and stimulation. THP-1 cells were obtained from the Korean Cell Line Bank (Seoul, Korea) and maintained in RPMI 1640 supplemented with 10% FBS, 1% penicillin-streptomycin and 0.1 mM of β-mercaptoethanol at 37 ℃ in humidified 5% CO_2 and 95% air. Differentiation of THP-1 cells was achieved by overnight stimulation with 100 nM phorbol-12-myristate-13-acetate (PMA). Cells were periodically tested for mycoplasma contamination. After PMA differentiation, the medium was replaced with Opti-MEM and cells were incubated for 1 h with or without BOT-4-one, KCl (150 mM), or zVAD (20 μM) before stimulation with nigericin (10 μM) for 1 h and ATP (5 mM) or silica crystal (150 μg/mL) for 3 h, and transfected with poly(dA:dT) (4 μg/mL) for 3 h using Lipofectamine 2000 (Invitrogen).

MTT assay. To determine the effect of BOT-4-one on cell viability, BMDMs and PMA-differentiated THP-1 cells were cultured in 96-well plates. The cells were treated with serially diluted BOT-4-one and incubated for 6 h. After treatment, the medium was discarded and MTT (0.5 mg/mL) was added to each well, followed by incubation for 4 h at 37 ℃. After incubation, the medium was discarded and DMSO was added to each well for the solubilization of formazan. Optical density was measured at 550 nM and values were calculated in comparison to the control cells.

Flow cytometric analysis. LPS-primed BMDMs were treated with BOT-4-one, and then stimulated with ATP or nigericin. Mitochondrial mass and membrane potential were measured by fluorescence level upon staining with Mitotracker green and Mitotracker deep red (Invitrogen, Life Technologies, Grand Island, NY, USA) at 50 nM at 37 ℃. Cells were then washed with PBS and resuspended in FACS buffer (0.2% FBS, 2 mM EDTA, 0.1% sodium azide in PBS) for FACS analysis. Samples were analyzed with FacsCalibur Flow cytometry (BD Pharmingen) and the data were analyzed using Cell Quest pro software (BD Pharmingen).
